# Dilemma Tales as African Knowledge Practice: An Example From Research on Obligations of Support

**DOI:** 10.3389/fpsyg.2020.546330

**Published:** 2020-10-02

**Authors:** Darlingtina Esiaka, Glenn Adams, Annabella Osei-tutu

**Affiliations:** ^1^Department of Psychology, Union College, Schenectady, NY, United States; ^2^Department of Psychology, University of Kansas, Lawrence, KS, United States; ^3^Department of Psychology, University of Ghana, Accra, Ghana

**Keywords:** African cultural models, dilemma tales, relationality, obligation, eldercare, family, interdependence

## Abstract

This contribution to the *Frontiers* research topic collection on *African Cultural Models* considers dilemma tales: an African knowledge practice in which narrators present listeners with a difficult choice that usually has ethical or moral implications. We adapted the dilemma tale format to create research tasks. We then used these research tasks to investigate conceptions of care, support, and relationality among participants in Ghanaian, African American, and European American settings that vary in affordances for embedded interdependence vs. modern individualism. Results revealed hypothesized patterns, such that judgments about the inappropriateness of institutionalized eldercare (vs. home elder care) and prioritization of material support to parent (over spouse) were greater among Ghanaian participants than European American participants. Responses of African American participants were more ambiguous, resembling European American acceptance of institutionalized eldercare relative to Ghanaian participants, but resembling Ghanaian tendencies to prioritize support to parent (over spouse) relative to European American participants. Results illuminate that standard patterns of hegemonic psychological science (e.g., tendencies to prioritize obligations to spouse over mother) are the particular product of WEIRD cultural ecologies rather than context-general characteristics.

## Introduction

A man was traveling with his mother and his fiancée. Their canoe was capsized by a sudden storm. The man was an excellent swimmer, but he could save only one woman. Which one should he choose? (Thomas, 1958–1959, p. 431).

This passage comes from a collection of what Bascom (1975) referred to as *dilemma tales*: a knowledge practice common across many African settings in which narrators present listeners with a difficult choice that usually has ethical or moral implications. The tales are an important form of informal socialization, teaching listeners not only about important cultural norms, but also moral reasoning skills.

*The content [of dilemma tales] is often didactic, but their special quality is that they train those who engage in these discussions in the skills of argumentation and debate and thus prepare them for participating effectively in the adjudication of disputes, both within the family or lineage and in formal courts of law* (Bascom, 1975, p. 1).

However, the tales also provide an important form of entertainment, especially for communities without easy access to radio, television, and other electronic media.

*[Dilemma tales] raise strangely difficult problems of morality and give rise to interminable palavers*… *This [literary form] reveals the cultural psychology, since it states precisely a very frequent type of social game and a highly prized kind of intellectual activity; one frequently sees groups discussing thus in the evening, not without passion and sometimes until a late hour in the night* (Thomas, 1958–1959, p. 431; as cited in Bascom, 1975, pp. 1–2).

### Dilemmas of Care

The particular dilemma tale that we cite at the beginning of this paper is one that has informed the work of our colleagues in the Cultural Psychology Research at the University of Kansas and collaborators at the University of Ghana (Salter and Adams, 2012; Esiaka, 2019; Osei-Tutu et al., 2020; Zhao et al., 2020). In this example, the listener must decide on an appropriate course of action for a person (as in the case of other dilemma tales, almost always a man) who in an emergency must choose to save either mother or fiancée, leaving the other to die. Although this particular example comes from Dyola communities in the Casamance region of Senegal, the basic structure of the dilemma is one that is popular across many African settings, the broader African diaspora, and perhaps beyond. Indeed, the immediate inspiration for investigations of the Cultural Psychology Research Group was not anything that Bascom (1975) recorded, but instead a calypso or mento song that featured prominently in the playlist of the house band at a jazz club in Accra^1^.

Bascom (1975) provides variations on the theme. One version from Lamba communities of present-day Zambia adds the person’s mother-in-law:

*A man, his wife, his mother, and his mother-in-law were attacked by a marauding band. They fled to the river, but his canoe could take only the man and one passenger. Which did he take?* (Doke, 1947, p. 119).

Another version from Bété communities of present-day Côte D’Ivoire exchanges the mother for the sister:

*In the course of crossing a river, a man’s canoe capsized, and he found himself in the water with his sister, his wife, and his mother-in-law. None of the women could swim. Which one should the man save?* (Paulme, 1961, p. 38; as cited in Bascom, 1975, p. 93).

As in these examples, the narrator of dilemma tales often ends with an explicit question or invitation to debate. In some cases, as in the example that we provide at the beginning of this paper, the narrator did not offer a resolution (Thomas, 1958–1959, p. 431; as cited in Bascom, 1975, p. 94). In the other examples that we take from Bascom’s (1975) collection, the narrators did provide a resolution. In the Bété tale, the narrator weighs the costs associated with different courses of action:

*If you save your sister and leave your wife to drown, you must give bride-wealth again [i.e., incur significant expense to acquire a new wife]. If you save your wife and abandon your sister, your parents overwhelm you with reproaches. But if you choose to save your mother-in-law, you are an idiot* (Paulme, 1961, p. 38; as cited in Bascom, 1975, p. 93; current authors added parenthetical explanation).

In the Lamba tale, Doke reports that “The solving provides much merriment. Of course NOT his mother-in-law; his wife then? No, he can get another wife! But he could not get another mother” (Doke, 1947, p. 119; as cited in Bascom, 1975, p. 93). This resolution closely resembles the musical version of the dilemma (e.g., The Jolly Boys, 1989).

I heard some foolish guys around the corner

Say that they’d rather their lawful wife than their mother.

I asked them the reason, and this they answered:

You can romance your wife but not your mother.

*My wife might be flourishing with gold and pearls*,

But my mother comes first in this blessed world.

*So I can always get another wife*,

But I can never get another mother in my life.

In summary, to the extent that the basic dilemma has a normative solution, responses of narrators across African (diasporic) settings suggest that one should prioritize care to mother over care to spouse. This normative solution appears to contrast with conventional wisdom in the WEIRD^2^ settings that disproportionately constitute the knowledge base of hegemonic psychological science. Both conventional and scientific wisdom in these settings dictate that people should prioritize obligations to mating connections over kinship connections. For example, prominent theoretical perspectives propose that mature adults should transfer their affective investment and associated basis for emotional security—what one might refer to as a primary attachment figure—from their parents to an intimate mating bond (e.g., Fraley and Davis, 1997; Feeney, 2004; Heffernan et al., 2012). From such perspectives, the tendency to prioritize connections to parent over the mating bond is a classic marker of unhealthy adjustment.

### Cultural Models of Relationality

The purpose of this contribution to the *Frontiers* research topic on *African Cultural Models* is to take the knowledge practice of the dilemma tale as an opportunity to consider cultural-ecological variation in experience of relationality. Embedded within different responses to the situation of these dilemma tales is a contrast between competing models of family and relationality. The approved solution for many African narrators—choosing mother or sister over spouse or fiancée—resonates with what Keller (2019) has referred to as a developmental pathway for hierarchical relationality attuned to cultural ecologies of embeddedness. These pathways prepare a person for competent action in accordance with the interdependent selfways (Markus et al., 1997) that are common across many African settings (Adams and Dzokoto, 2003). Although specifics vary according to details of particular cultural ecologies—including subsistence practices (Edgerton, 1971), kinship practices (Lowes, 2020), religious engagement (Osei-Tutu et al., 2020), or rural/urban residence (Adams and Plaut, 2003; Salter and Adams, 2012)—everyday realities in many African settings afford an experience of self in terms of inherent connection not only to living people, but also to ancestors, spiritual entities, land, and material realities of place (Adams and Dzokoto, 2003).

These constructions of self and society often find expression in local aphorisms or proverbs. A particularly illuminating example is the Kuranko (Sierra Leone) idea that “One’s birth is like the bird-scaring rope” (Jackson, 1982, p. 17).

The bird-scaring rope is an agricultural tool that consists of a network of rope with bits of metal tied to stakes and stretched back and forth across a field. By tugging from a central point, a farmer sets in motion the whole network of rope and produces a cacophony of clanking metal that scares marauding birds away from the maturing rice crop. This tool serves as an apt metaphor for personal experience in many West African settings. People emphasize that they do not exist in isolation; rather, their actions … trigger consequences for others that reverberate across networks of social relations like a tug of the bird-scaring rope (Adams and Kurtiş, 2018; pp. 164–165).

Adaptive functioning in cultural ecologies of embeddedness and interdependence requires coordination of activity—and often the subordination of personal aspirations—to ensure broader collective viability.

The contrasting solution—choosing spouse or fiancée over mother or sister—resonates with what Keller has referred to as developmental pathways for psychological autonomy (and psychological relatedness) attuned to cultural ecologies of modern individualism. These pathways prepare a person for competent action in accordance with independent selfways (Markus et al., 1997): an experience of self in terms of defining psychological attributes abstracted from social and physical context (Adams et al., 2019). Associated with these selfways is a construction and experience of relationship as a secondary product of ontologically prior individuals—connections that people feel free to build or abandon depending on their promise to deliver personal fulfillment and satisfaction (Adams et al., 2004).

### Implications for Conceptions of *Family*

Cultural ecologies of embeddedness and interdependence promote a relatively broad construction and experience of family as lineage: the pre-existing and enduring kinship reality in which the events of life—birth, mating, parenthood, and death—take place. As even a brief survey of ethnographic literature reveals, kinship systems vary widely across African settings, with some societies defining family belonging and residence according to maternal relations, others according to paternal relations, and others some combination of the two (Radcliffe-Brown and Forde, 1950/1987; Guyer, 1981). Across this diversity of forms, a unifying idea is conception of kinship as something natural, enduring, built into the social order, more central to a person’s being, and more prominent in psychological experience than artificial and tenuous relationships like marriage. Accordingly, conventional wisdom in many African settings is that people should trust and prioritize obligations to kinship connections over mating connections (e.g., Fortes, 1950/1987). More generally, research suggests a similar pattern of relative preference for mother over spouse in other Majority-World settings, outside WEIRD enclaves, where interdependent selfways are prominent (Wu et al., 2016; Zhao et al., 2020).

Again, this construction and experience of family contrasts with modern individualist understandings typical of WEIRD realities that disproportionately inform hegemonic psychological science. These cultural ecologies promote a relatively narrow construction of *family* as the nuclear household centered around mating unit or “the sacred couple” (Shweder et al., 1995; see Adams et al., 2004). Reflecting the modern individualist understanding of relationship as the secondary creation of ontologically prior individuals, these cultural ecologies promote a construction of kinship as something like an artificial imposition, and the more consequential relationships are ones that people choose and voluntarily create. This is not to deny that people who inhabit WEIRD settings and cultural ecologies of modern individualism form intimate connections in ascribed relationships with parents and siblings; instead, the point is that people choose to invest in these relationships. They do not feel obliged to do so merely by virtue of some accident of birth, but invest, or not, as a function of their satisfaction with the relationship (Miller, 1994). Accordingly, conventional wisdom in these spaces is that people should trust and prioritize obligations to mating partners over kinship connections. More generally, a defining feature of modern individualist subjectivity is devaluation of traditional familial authority in favor of individual rational choice (Inkeles, 1969).

### The Present Study

Our review of African dilemma tales suggests a prioritization of care to mother over spouse that appears to be at odds with conventional wisdom in hegemonic psychological science (e.g., prescribing a transfer of attachment from parent to mating partner; Fraley and Davis, 1997; Feeney, 2004; Heffernan et al., 2012). We have explained this difference in prioritization as a function of differences in the construction and experience of relationship associated with different selfways. Our empirical work suggests that these differences are not limited to collective discourse, but also appear in the responses of individual research participants to whom we have presented different adapted versions of this dilemma. In some cases, we have asked participants to respond to a task—choosing to save either mother or spouse—that is more or less as the narrator of the dilemma tale stated it (Osei-Tutu et al., 2020). In other cases, we have adapted the dilemma by asking participants to allocate scarce resources for healthcare between mother and spouse (Salter and Adams, 2012). Faced with dilemmas involving allocation of scarce care resources, people in a variety of West African settings—but especially outside large cities—tend to prioritize care to mother over spouse (Salter and Adams, 2012; Esiaka, 2019; Osei-Tutu et al., 2020). This pattern contrasts with responses of participants in a variety of settings in the United States, who tend to respond to the same research tasks by prioritizing care to spouse over mother (Salter and Adams, 2012; Wu et al., 2016; Esiaka, 2019; Zhao et al., 2020). The current study extends our previous research in four ways.

#### Navigating Competing Requests for Financial Assistance

First, one critique of research using the original dilemma tale is that it presents participants with an extraordinary emergency that may reveal little about their performance of more ordinary obligations for support. In the current study, we use an adaptation of the dilemma in which participants must decide how to respond to competing request for financial assistance.

#### Obligations to Children

Second, we extend the typical focus on allocation of assistance to mother or spouse by adding a third choice where obligations of care are perhaps greatest: that is, child. Children occupy a theoretically ambiguous position in the structure of the original dilemma. On one hand, children are similar to mother in that they are kin; on the other hand, children are similar to spouse in that they are a product of connections that one has chosen. The current study explores the implications for dilemma responses when a respondent must allocate financial assistance between elderly parent, spouse, and young child.

#### Eldercare Dilemma

Third, we extend our previous research by adding a dilemma associated with the contemporary condition of life in the Eurocentric modern global order. Specifically, we ask participants to consider two courses of action regarding care of an elderly parent.

One course of action that resonates with the bureaucratic efficiency aspect of modern individualist selfways (e.g., Inkeles, 1969) is institutionalization in a care facility. A benefit of this solution is to afford maximum independence of both elderly parent and adult child. Ideally, life in a care facility affords the elderly parent the opportunity for satisfying interactions with peers from the same age cohort, with whom they share generational common ground. At the same time, institutional care allows the elderly parent to avoid invasions of their privacy, undesirable dependence on children, or impositions on the child’s household. From the child’s perspective, institutionalization ideally can provide reassurance that the parent is receiving competent care and other kinds of attention that the child might be unable to provide. Equally important, institutionalization can allow the adult child to avoid strain on relationships with and obligations to partner and children.

The contrasting course of action is integration of the elderly parent into the nuclear family household of the adult child. This course of action resonates with the interdependent selfways associated with realities of embeddedness and hierarchical relatedness. This solution prioritizes enduring intergenerational obligations and connections between relatives over the more disembedded and voluntaristic relations of cohort mates (in the case of elders) and spouse (in the case of adult child).

#### African American Settings

Finally, we extend our previous research by extending our comparison to a third group of participants: people who claim African American identity. To some extent, the theoretical importance of this extension concerns the typical interest in “bicultural” experience of people who occupy spaces somewhere in between groups constituting a “cross-cultural” comparison (i.e., *African* and *American*). More generally, this extension has potential to inform longstanding discussions in African American Studies concerning the origins of distinctive cultural-psychological tendencies. Although a thorough discussion is beyond the scope of the current work, scholars in African American studies have debated the extent to which one should understand distinctive cultural psychological tendencies of people in African American communities as either the enduring cultural influence of African traditions, adaptations to conditions that people of African descent have faced in the U.S. (especially including the horrific violence of enslavement and societal racism), or both (Boykin and Toms, 1985; Sudarkasa, 2007).^3^ Indeed, one of the primary themes of Bascom’s (1975) work was that African American folktales were not simply adaptations of European stories, but instead had roots in African cultural sources. In this respect, he followed in the footsteps of his mentor, Melville Herskovits (e.g., Herskovits, 1941), who was a leading (albeit complicated) proponent of ideas about enduring African influences in African American cultural patterns. In the current study, we consider the extent to which African American responses resemble African or European American patterns.

## Materials and Methods

### Participants

We recruited European American (EA: *N* = 153; 50.3% men; ages 21–72, *M* = 34.50, *SD* = 11.39) and African American (AA: *N* = 105; 45.7% men; ages 20–64, *M* = 30.95, *SD* = 7.96) participants from Amazon Mechanical Turk (MTurk), an online marketplace where people take part in academic research for a fee. We recruited Ghanaian participants (GH: *N* = 163; 67.5% men; ages 18–70, *M* = 30.71, *SD* = 11.44) from Legon, a university community within Accra, the cosmopolitan, capital city of Ghana. We aimed to recruit participants who are community-dwelling adults, not undergraduate students, and at the age where they have made decisions about eldercare or have extensive knowledge of eldercare provision.

### Materials and Procedure

The work we report here was part of a larger investigation of social relations in West African and North American settings. Details about the larger project are available from the first author. Participants in Ghana completed a paper version of the survey. Participants from the US completed an online version of the survey.

Participants read descriptions about two scenarios involving care decisions. We presented the scenarios in a way that resembled the West African genre of a dilemma tale (Bascom, 1975).

#### Care of Elder: Integration or Institutionalization?

The first dilemma involved a choice of action about care for an aging parent. Participants read the following description:

Alex is a 40-year adult, married with two (2) children. One of Alex’ parents recently died after a heart attack. The other parent, who lives alone in a town that is two (2) hours away, has become increasingly frail, and requires assistance to manage everyday life tasks.

Participants used a 5-point Likert scale ranging from -2 (completely disagree) to 2 (completely agree) to indicate their agreement with two options for resolving the dilemma. The first option was integration of the parent into the home: “When an elderly parent can no longer live alone, the best solution is for an adult child to invite the parent to stay in child’s home.” The second option was institutionalization in an elder care facility: “When an elderly parent can no longer live alone, the best solution is for the parent to move to an assisted living facility or eldercare community.”

#### Competing Claims for Material Support

The second dilemma involved a decision about allocation of scarce financial resources between three potential recipients. Participants read the following description:

Alex is a 45-year professional who faces a problem of competing obligations. The retired parents have asked for financial assistance to help them with repairs to Alex’s childhood home, where they have lived for the last 50 years. The spouse (also a professional) has asked for financial assistance to address a loan repayment issue. The eldest of two children has gained acceptance to a prestigious university and has asked for financial assistance to cover tuition costs.

Participants then indicated the percentage of available funds they would “advise Alex to allocate to each person?”

After completing the two tasks, participants responded to several demographic questions regarding their age, gender, ethnicity, and marital status. The participants indicated their subjective social standing by choosing where they perceive themselves to stand on a ten-rung ladder (from 1 = *worst off* to 10 = *best off*) that represented everyone in the society. They also indicated number of living parents, whether they were raised by an elder, and whether they were currently co-resident with a parent.

## Results

A summary of demographic information appears in Table 1. European American participants were older on average than both African American and Ghanaian participants (Table 1). More important for current purposes, the proportion of participants who (a) lived in close proximity with an elder as a child or (b) were co-resident with parents at the present time was greater among Ghanaian participants (38 and 37%, respectively) than European American participants (12 and 6%, respectively), χ^2^ = 27.41, *p* < 0.001. In contrast, the proportion of participants who were married or living as conjugal partners was greater among European American participants (50%) than among Ghanaian participants (29%), χ^2^ = 16.72, *p* < 0.001. Responses of African American participants were intermediate between responses of these two groups (27, 11, and 38%, respectively). These patterns of everyday life constitute evidence for the theoretical framework that we have articulated in the introduction. Consistent with this framework, conjugal partnerships figure more prominently, and relations with elders figure less prominently, in the everyday cultural ecology of European American worlds than in the everyday cultural ecology of Ghanaian worlds.

**TABLE 1:** Demographic and social characteristics across research settings.

	Ghanaian	African American	European American

	*N* = 163	*N* = 105	*N* = 153
Gender (% Male)	68	46	50
Age	30.7 (11.4)	31.0 (8.0)	34.5 (11.4)
Education (more than 12 years)	91	95	96
Marital Status (% married)^*a*^	29	38	50
Subjective social standing	5.3 (1.8)	4.7 (1.7)	4.8 (1.8)
Both parents alive (%)	62	69	69
Parental co-residence status (%)	37	11	6
Raised by an elder (%)	38	27	12

**^*a*^Married includes unmarried adults living as married.**

Although these patterns are consistent with our theoretical framework, they complicate interpretation of evidence regarding dilemma choices. For example, patterns of correlation (Table 2) indicate that being raised by an elder and present co-residence with an elderly parent were positively related to the dilemma response of allocation to parent. This raises the possibility that any differences in dilemma choices across settings may be a product of different life circumstances (a history of residence with elders) rather than the more embodied or internalized prioritization of spouse or parent. Although this would not invalidate our primary point about cultural-ecological variation in dilemma responses, we nevertheless explored this possibility by conducting supplementary analyses in which we controlled for demographic variables by including them as covariates in analyses that we report below. Results of these analyses do not alter interpretations. Details of these analyses are available from the first author.

**TABLE 2:** Correlation matrix of key constructs among all samples.

	2	3	4	5	6	7	8	9	10	11
1. Integration	−0.05	0.59**	0.04	0.03	−0.08	0.11*	0.14**	0.02	0.12*	−0.04
2. Institutionalization		−0.58**	−0.06	0.06	0.01	0.05	0.05	0.06	−0.03	−0.07
3. Preference for integration		–	−0.01	−0.02	0.02	0.06	0.03	0.04	0.05	0.02
4. Allocation to parent			–	−0.35**	−0.53**	−0.10*	−0.05	−0.08	0.17**	0.14**
5. Allocation to spouse				–	−0.57**	0.12*	0.07	−0.02	−0.11*	−0.12*
6. Allocation to child					–	−0.03	−0.04	0.09	−0.05	−0.01
7. Age						–	0.34**	−0.05	−0.05	0.03
8. Marital status							–	0.13**	0.02	−0.00
9. Social standing								–	0.06	0.19**
10. Raised by an elder									–	0.27**
11. Parent coresident										–

**p < 0.01, *p < 0.05.

### Eldercare Dilemma

To analyze the results for the first dilemma, we conducted a 3 × 2 mixed-model Analysis of Variance (ANOVA) with Cultural Setting (Ghanaian, African American, and European American) as the between-participants factor and Degree of Integration (*arrange for care facility* or *bring to home*) as the within-subject factor. Results revealed a main effect of Degree of Integration on responses to the eldercare dilemma, *F*(1, 414) = 14.15, *p* < 0.001, η*_*p*_*^2^ = 0.03. As indicated in Table 3, participants judged the high-integration response (*bring to home*: *M* = 0.47, *SD* = 1.14) as a better solution than the low-integration response (*arrange for care facility*: *M* = 0.14, *SD* = 1.12). More important for present purposes, results revealed an anticipated interaction of Cultural Setting X Degree of Integration, *F*(2, 414) = 3.95, *p* = 0.02, η*_*p*_*^2^ = 0.02. To probe the interaction, we conducted one-way ANOVAs to examine differences in agreement with each response across cultural settings. There was no difference across settings in agreement with the high integration response, *F*(2, 418) = 0.75, *p* = 0.47, η*_*p*_*^2^ = 0.00, but there was a significant difference in agreement with the low integration response, *F*(2, 414) = 8.15, *p* = 0.00, η*_*p*_*^2^ = 0.04. Pairwise comparisons using the Tukey procedure confirmed significant differences such that American participants indicated approval of the institutionalization option (African American: *M* = 0.33, *SD* = 1.09; European American: *M* = 0.31, *SD* = 1.10), but Ghanaian participants tended to express disapproval of this option (*M* = -0.15, *SD* = 1.26).

**TABLE 3:** Dilemma responses across research settings.

	Ghanaians	African Americans	European Americans
**Variable**			
Eldercare best solution			
Integration	0.42 (1.33)_*a*_	0.57 (0.93)_*a*_	0.41 (1.04)_*a*_
Institutionalization	−0.15 (1.26)_*a*_	0.33 (1.09)_*b*_	0.31 (1.10)_*b*_
Preference for Integration (%)	55.3	40.0	37.3
Allocation of funds			
Spouse	31.09 (9.60)_*a*_	31.91 (14.57)_*a*_	37.04 (14.48)_*b*_
Child	40.72 (12.34)_*a*_	38.29 (16.13)_*a*_	36.92 (15.01)_*a*_
Parent	28.36 (11.26)_*a*_	29.59 (13.90)_*a*_	25.98 (13.25)_*a*_

**Within each row, means with different subscripts differ significantly using Tukey procedure (p = 0.05). Numbers in parenthesis are standard deviations.**

Another way to probe the interaction—one that resonates better with the dilemma tale genre—is to transform rating responses into a categorical *preference for integration* variable that indicates whether participants expressed greater agreement with the high integration, *bring to home* option than the low integration, *arrange for care facility* option. Consistent with hypotheses, the proportion of participants whose responses implied this preference for integration of elder into the home was greater for Ghanaian participants (55.3%) than for African American (40.0%) or European American participants (37.3%), χ^2^(2) = 11.65, *p* = 0.003.

### Material Support Dilemma

To analyze the results for the second dilemma, we conducted a 3 × 3 mixed-model ANOVA with Cultural Setting (Ghanaian, African American, and European American) as the between-participants factor and Relationship (Child, Spouse, Parents) as the within-subject factor. Results revealed a main effect of relationship, such that participants tended to prioritize support to child (*M* = 38.72; *SD* = 14.42) over spouse (*M* = 33.47; *SD* = 13.11) or parent (*M* = 27.80; *SD* = 12.76), *F*(2, 430) = 43.53, *p* < 0.001, η*_*p*_*^2^ = 0.10. More important for present purposes, results revealed an anticipated interaction of Cultural Setting X Relationship, *F*(2, 430) = 4.89, *p* = 0.001, η*_*p*_*^2^ = 0.02. To probe the interaction, we conducted one-way ANOVAs to examine differences in allocation to each relationship across cultural settings. Results indicated significant differences for allocation to spouse, *F*(2, 417) = 9.30, *p* < 0.001, η*_*p*_*^2^ = 0.04, but only marginally significant for allocation to parent, *F*(2, 417) = 2.80, *p* = 0.06, η*_*p*_*^2^ = 01. and child [*F*(2, 417) = 2.80, *p* = 0.06, η*_*p*_*^2^ = 0.01]. Information about significant pairwise comparisons using the Tukey procedure appears in Table 2.

A narrative summary of differences across settings is this. Whereas participants in all settings tended to prioritize support to child (*M*s > 36.92; *SDs* > 12.33), participants differed across settings in prioritization of support to spouse vs. parent. European Americans tended to allocate as much to spouse (*M* = 37.04; *SD* = 14.48) as they did to child, at the expense of parent (*M* = 25.98; *SD* = 13.35), a difference of 11.06, *t*(152) = 5.85, *p* < 0.001, *d* = 0.79. In contrast, allocations of support to spouse tended to be smaller and less different than allocations to parent for Ghanaians (spouse: *M* = 31.09, *SD* = 9.60; parent: *M* = 28.36; *SD* = 11.26; difference = 2.73), *t*(159) = 2.73, *p* = 0.03, *d* = 0.26, and African Americans (spouse: *M* = 31.91, *SD* = 14.56; parent: *M* = 29.59; *SD* = 13.90, difference = 2.32), *t*(159) = 1.01, *p* = 0.31, *d* = 0.16.

## Discussion

Theory suggests, and previous research has found, that adult participants in WEIRD settings respond to adaptations of the mother-spouse dilemma tale by prioritizing assistance to spouse over assistance to mother (Salter and Adams, 2012; Wu et al., 2016). Results of the present study replicate this pattern in a novel adaptation of the dilemma tale that adds claims for assistance from a child alongside claims from mother and spouse. Although participants across settings tended to prioritize claims of the child, European American participants prioritized claims from a spouse to a greater degree than did Ghanaian participants. Indeed, European American participants prioritized claims from a spouse to the same extent that they did claims from a child, at the expense of claims from mother. In contrast, after prioritizing care to child, Ghanaian participants tended to allocate remaining resources more equally between spouse and mother.

Besides replicating patterns of previous research in an extended adaptation of the mother-spouse dilemma tale, results of the present study extend this pattern to a new dilemma involving care of an elderly parent. Participants across settings tended to evaluate integration of an elderly parent into the home as a better option than housing the parent in an elder care institution. However, consistent with theoretical expectations, and mirroring the pathways for psychological autonomy associated with modern individualist selfways, European American participants tended to evaluate institutional care of elders more favorably than did Ghanaian participants, who tended to express outright disapproval of this option.

Our data illustrate how variations in ideas about family and kinship across cultural settings promote particular understanding of interpersonal obligations. Thinking from the standpoint of West African epistemic locations illuminates the culture-bound character of the WEIRD inspired construction of family and relationality. Our findings support earlier research that construction of family and relationality in WEIRD settings de-emphasizes obligation to consanguine family (e.g., Adams et al., 2012; Salter and Adams, 2012; Esiaka, 2019). Cultural settings of embedded interdependence afford a duty-based interpersonal relationality that stresses broad and socially enforceable interpersonal obligations (e.g., Nsamenang and Lamb, 1995; Miller, 1997; Salter and Adams, 2012; Evans, 2015; Atakere and Adams, 2018; Esiaka, 2019; Zhao et al., 2020).

### African American Patterns

The present study also extends previous work by considering a novel cultural setting, participants who claim African American identity. The question of interest is whether responses of these participants would look more like European American patterns or Ghanaian patterns. Results of the present study are ambiguous.

On one hand, with respect to the adaptation of the mother-spouse dilemma tale, results for African American participants resemble results for Ghanaian participants. Both African American and Ghanaian participants tended to allocate resources more-or-less equally between spouse and parent. They did not show the prioritization of spouse over parent that European American participants showed. In this way, results are consistent with (but in no way prove) the idea of enduring African cultural influence on African American psychological patterns.

On the other hand, with respect to the dilemma involving care to an elder parent, results for African American participants resemble results for European American participants. Perhaps reflecting their common experience of U.S. institutional realities, both African American and European American participants tended to evaluate institutional care of elders more favorably than did Ghanaian participants. They did not express the same outright disapproval of institutionalization that Ghanaian participants showed.

Here again, we must leave the explanation for these ambiguous results as a topic for future research. We simply note that this ambiguity is consistent with the “in-betweenness” associated with bicultural settings. To the extent that African American settings are bicultural—somewhere between African and (European) American cultural worlds—one can anticipate that responses of African American participants will be intermediate between responses of Ghanaian and European American participants. Although results for the two tasks considered in isolation do not provide strong evidence of intermediacy—that is, African American participants resemble either Ghanaians or European Americans, rather than occupy an intermediate position between the two—the ambiguity of results across the two tasks taken together is consistent with it^4^.

### Limitations and Future Directions

As with any study, our investigation is not without limitations. On the independent variable side, although our interest is cultural-ecological affordances for modern individualism and embedded interdependence, we did not measure these affordances directly. Instead, we conducted a comparison of existing groups of people who inhabit Ghanaian and U.S. settings that previous work has associated with differences on these dimensions (e.g., Adams and Dzokoto, 2003; Adams and Plaut, 2003; Adams, 2005; Adjei, 2019). Moreover, even if observed differences in reactions to dilemmas are a function of affordances for embeddedness and modern individualism, the reliance on existing groups cannot address questions about the distal causes of observed differences in these affordances. Still, we believe that comparison of existing groups serves a useful function by illuminating the extent to which standard patterns of hegemonic psychological science—such as tendencies to prioritize obligations to spouse over mother—are the particular product of WEIRD cultural ecologies rather than context-general characteristics. This recognition is a necessary step toward decolonizing psychological science and reconstructing it in ways that resonate with Majority World experience (e.g., Adams et al., 2015).

On the dependent variable side, the study considered particular dilemmas with features that may not generalize to other care decisions or ways of asking the question. We address this limitation in other work in which we observe similar patterns of results using different adaptations of the dilemma tale (Salter and Adams, 2012; Esiaka, 2019; Osei-Tutu et al., 2020). Still, one should exercise restraint before overinterpreting results of this particular investigation.

A particularly important limitation concerns gender dynamics. We investigated, but did not observe significant differences, as a function of participant gender. However, we did not consider the possible effects of the target elders’ gender. Rather than systematically vary the gender of elders in the dilemma scenarios, we referred to gender-neutral parents. In other work, we have held parent gender constant by asking participants to respond with respect to mother (e.g., Salter and Adams, 2012; Osei-Tutu et al., 2020). An interesting direction for future research will be to examine more carefully differences in interaction with mothers and fathers, both within and across settings.

## Conclusion: Dilemma Tale as Indigenous Knowledge Practice

Our research task asked participants to consider a hypothetical dilemma involving another person. On one hand, a limitation of this procedure is that one cannot interpret responses as an indication of what the participants themselves would do when faced with the situation. On the other hand, a strength of this procedure is fidelity to the dilemma tale genre, which poses a question in terms of another person and then asks participants how the actor should, or how they would, respond in that situation. In the context of this Frontiers Research Topic, one might understand the genre of dilemma tale, like that of proverbs (Dzokoto et al., 2018), as an African Cultural Model for knowledge, theory, and research practice. Our work suggests that dilemma tales are important as repositories of cultural knowledge (e.g., about relationality), as pedagogical tools, and as an empirical method for investigations of moral reasoning and other psychological tendencies.

## Data Availability Statement

The raw data supporting the conclusions of this article will be made available by the authors, without undue reservation, to any qualified researcher.

## Ethics Statement

The studies involving human participants were reviewed and approved by the Human Subjects Committee Lawrence Campus (HSCL), University of Kansas. The participants acknowledged the information statement to participate in this study.

## Author Contributions

DE contributed to the study design, data collection, data analyses and interpretation, and writing of the manuscript. GA contributed to the study design, data analyses and interpretation, and writing of the manuscript. AO-t contributed to data collection and data preparation. All authors contributed to the article and approved the submitted version.

## Conflict of Interest

The authors declare that the research was conducted in the absence of any commercial or financial relationships that could be construed as a potential conflict of interest.

## Footnotes

1 For a similar recorded version of the number, consider Jamaican artists, The Jolly Boys (1989). Versions of the dilemma tale also circulated widely on the internet during the decade of the 2010s.

2 We borrow this acronym as an apt characterization for the disproportionate basis of mainstream psychological science in settings that are Western, educated, industrial, rich, and (supposedly) democratic (Henrich et al., 2010).

3 For example, researchers have noted a relative prevalence of female-run households in African American communities. While some observers (perhaps most infamously Moynihan, 1965) have explained female-run households as an adaptation to conditions in the Americas, others have explained it as an enduring cultural influence of African family systems (Nobles, 2006; Carson, 2009).

4 Exploratory analyses of correlations involving sociodemographic indicators—for example, age or SES—did not reveal any significant relationships that might explain variation in dilemma responses of African American participants. Details of these analyses are available from the first author.
